# Structural Basis for Binding of Fluorinated Glucose and Galactose to *Trametes multicolor* Pyranose 2-Oxidase Variants with Improved Galactose Conversion

**DOI:** 10.1371/journal.pone.0086736

**Published:** 2014-01-21

**Authors:** Tien Chye Tan, Oliver Spadiut, Rosaria Gandini, Dietmar Haltrich, Christina Divne

**Affiliations:** 1 Department of Medical Biochemistry and Biophysics, Karolinska Institute, Stockholm, Sweden; 2 School of Biotechnology, Royal Institute of Technology, Stockholm, Sweden; 3 Food Biotechnology Laboratory, BOKU University of Natural Resources and Life Sciences, Vienna, Austria; Institute of Enzymology of the Hungarian Academy of Science, Hungary

## Abstract

Each year, about six million tons of lactose are generated from liquid whey as industrial byproduct, and optimally this large carbohydrate waste should be used for the production of value-added products. *Trametes multicolor* pyranose 2-oxidase (*Tm*P2O) catalyzes the oxidation of various monosaccharides to the corresponding 2-keto sugars. Thus, a potential use of *Tm*P2O is to convert the products from lactose hydrolysis, D-glucose and D-galactose, to more valuable products such as tagatose. Oxidation of glucose is however strongly favored over galactose, and oxidation of both substrates at more equal rates is desirable. Characterization of *Tm*P2O variants (H450G, V546C, H450G/V546C) with improved D-galactose conversion has been given earlier, of which H450G displayed the best relative conversion between the substrates. To rationalize the changes in conversion rates, we have analyzed high-resolution crystal structures of the aforementioned mutants with bound 2- and 3-fluorinated glucose and galactose. Binding of glucose and galactose in the productive 2-oxidation binding mode is nearly identical in all mutants, suggesting that this binding mode is essentially unaffected by the mutations. For the competing glucose binding mode, enzyme variants carrying the H450G replacement stabilize glucose as the *α*-anomer in position for 3-oxidation. The backbone relaxation at position 450 allows the substrate-binding loop to fold tightly around the ligand. V546C however stabilize glucose as the *β*-anomer using an open loop conformation. Improved binding of galactose is enabled by subtle relaxation effects at key active-site backbone positions. The competing binding mode for galactose 2-oxidation by V546C stabilizes the *β*-anomer for oxidation at C1, whereas H450G variants stabilize the 3-oxidation binding mode of the galactose *α*-anomer. The present study provides a detailed description of binding modes that rationalize changes in the relative conversion rates of D-glucose and D-galactose and can be used to refine future enzyme designs for more efficient use of lactose-hydrolysis byproducts.

## Introduction

Bioconversion of inexpensive and abundant resources into added-value products is highly desirable for a sustainable development. The disaccharide lactose is a significant byproduct produced in large quantities by the dairy and whey industries. For instance, some six million tons of lactose are generated each year from liquid whey alone [1]. Lactose is easily degraded to D-glucose (Glc) and D-galactose (Gal) by the enzyme β-galactosidase (EC 3.2.1.23). To further convert D-glucose and D-galactose to more valuable sugar compounds, an enzyme equally efficient in converting both substrates would be ideal. In this context, *Trametes multicolor* pyranose 2-oxidase (*Tm*P2O; EC 1.1.3.10 [2]; for a review, see [3]) is of interest since it can oxidize both *β*-D-Glc and *β*-D-Gal to the corresponding 2-keto sugars [4], which are intermediates for production of D-fructose and D-tagatose [5]. However, catalytic turnover of these sugars by wild-type *Tm*P2O is highly biased towards Glc, with only minor Gal being oxidized [6]. Ideally, the enzyme should convert the sugars at near equal rates, and with considerable efficiency.

*Tm*P2O is a homotetrameric enzyme produced by lignocellulolytic fungi and has been assigned a role in lignin degradation [7]–[11]. The crystal structure of a pyranose 2-oxidase was first determined in 2004 from *Trametes multicolor* [12], and from *Peniophora sp*. [13]. Since then, we have studied various aspects of *Tm*P2O with particular emphasis on the mechanism of regioselectivity and the importance of active-site loop conformational dynamics for substrate specificity using a combination of rational and semi-rational protein design, biochemical characterization and crystal structure analysis [14]–[19]. We have also reported the crystal structure of P2O from *Phanerochaete chrysosporium* [20], as well as of the related flavoprotein subunit of cellobiose dehydrogenase [21], [22], pyranose dehydrogenase from *Agaricus meleagris* [23]; all of which are members of the glucose-methanol-choline (GMC) family of large oxidoreductases [24], [25].

The reaction catalyzed by *Tm*P2O is of the ping-pong type [26] involving a reductive and an oxidative half-reaction. During the reductive half-reaction, *β*-D-Glc is oxidized at C2 to 2-keto-D-Glc [6], [26] with the concomitant reduction of the flavin adenine dinucleotide (FAD) to FADH_2_. Regioselectivity of the reaction with Glc is high, resulting in only 2-oxidation [8], [19], [27], [28]; however, oxidation at C3 has been reported for some substrates [27]. After release of the first product (keto sugar), the substrate of the oxidative half-reaction, O_2_, is reduced to H_2_O_2_ and the flavin re-oxidized to the ground state, and primed for a new redox cycle. The mechanism of the oxidative half-reaction proceeds *via* the pH-dependent formation of a transient covalent flavin C(4a)-hydroperoxide intermediate [29], [30].

The *Tm*P2O variant H167A lacks the covalent bond between His167 and the flavin C8α atom [14], and has proved to be an invaluable model enzyme for crystal-structure analysis of substrate-binding modes in *Tm*P2O due to: *(i)* its significantly lower rate of flavin reduction compared with the wild type; *(ii)* its ability to catalyze the oxidation of Glc and Gal regioselectively at C2 during the reductive half-reaction; and *(iii)* it displays a behavior similar to that of the wild-type *Tm*P2O in the oxidative half-reaction [14], [28]. Thus, in all important aspects this variant serves the purpose of a wild-type mimic, but with the advantage of slower sugar oxidation [14], [19]. Compared with the wild type, the H167A variant displays more complex binding kinetics, suggesting that two binding pathways exist that merge to form a single enzyme-substrate complex [28]. It has been suggested that the multiple binding pathways may arise because of increased flexibility of the non-covalently bound FAD leading to additional spatial freedom below the flavin ring to accommodate additional binding modes of Glc [28].

Based on the high-resolution crystal-structure analysis of H167A in complex with Glc fluorinated at C3 (3-deoxy-3-fluoro-D-Glc; *3F*Glc), we recently described the productive 2-oxidation binding mode for Glc in detail (PDB code 3PL8 [19]). Using Glc fluorinated at C2 (2-deoxy-2-fluoro-D-Glc; *2F*Glc) allowed us to identify and describe a possible competing binding mode, *i.e*., Glc oriented for oxidation at C3 (PDB code 2IGO [14]). That both the 2- and 3-oxidation binding modes are relevant is evidenced by reduction experiments showing that fluorinated sugars are indeed slow substrates, and not inhibitors [14].

We expect that for any given substrate, different competing binding modes will exist, but only one will result in a desired catalytic event of productive reaction outcome. The relative binding affinities for any nonproductive binding mode will affect the overall efficiency of catalytic turnover. At the structural level, we define the productive binding mode for Glc according to three main criteria: *(i)* the sugar is oriented for oxidation at C2 [14], [19]; *(ii)* the substrate-binding loop is in the semi-open conformation [19]; and *(iii)* the side chain Oγ1 group of Thr169 is pointing *away* from the flavin N(5)/O(4) locus, *i.e.,* it does not form hydrogen bonds with either N(5) or O(4) [19], [31].

In the case of Glc oxidation, the sugar orientation that may compete with the productive 2-oxidation binding mode corresponds to the nearly isosteric 3-oxidation mode, a mode that is stabilized by an almost identical set of protein-sugar interactions. Thus, when Glc is concerned, the sugar binding modes for 2-oxidation [19] and 3-oxidation [14] are indeed very similar. The active site in *Tm*P2O is spatially restricted, and the two binding modes are related by a 180° “upside-down” rotation of the pyranose ring, a transformation that would be impossible while the sugar is still bound to the enzyme. Therefore, in the event of initial binding of a Glc molecule in the competing orientation for 3-oxidation, the only possibility to achieve productive oxidation at C2 is that the active site expels the sugar, followed by a new binding attempt, possibly placing it correctly for 2-oxidation. As pointed out above, the enzyme may under certain circumstances even catalyze oxidation at C3 [27].

Under normal conditions, however, the wild-type enzyme ensures high regioselectivity for 2-oxidation [8], [19], [27], [28] by fine-tuning the conformation of the substrate-recognition loop, and the precise and relative position of a set of core amino-acid side chains in the loop and in the active site [19]. We proposed a mechanism for the regioselective oxidation of Glc by *Tm*P2O where Asp452, Tyr456 and Arg472 were pointed out as critical residues for discrimination between the two competing binding modes of Glc [12], [14], [19], *i.e*., between the mode where Glc is oriented for productive oxidation at C2 (represented by *3F*Glc), and the competing nonproductive binding mode where the Glc C3 atom (represented by *2F*Glc) is occupying the oxidation site.

With the aim to identify *Tm*P2O variants with improved turnover of Gal and a more equal conversion of Glc and Gal, results from saturation mutagenesis returned the *Tm*P2O variant H450G as having increased Gal conversion rates with O_2_ as electron acceptor compared with the wild type, while retaining acceptable Glc conversion, giving a lower Glc-to-Gal selectivity [32]. His450 is located immediately before the start of the highly dynamic substrate-recognition loop in *Tm*P2O (residues 451–461), but is itself not part of the conformationally more dynamic region of the active site [12], [14], [18], [19]. Another *Tm*P2O mutant of interest is V546C, where increased turnover rates for both Glc and Gal were observed, but with increased *K*_m_ values [15], [33]. However, the overall Glc-to-Gal selectivity ratio for the V546C mutant was increased relative to the wild type. By combining the two aforementioned mutations, H450G and V546C, a third enzyme variant was investigated that showed increased Gal conversion and decreased turnover of Glc accompanied by slightly increased binding constants for both substrates, leading to a Glc-to-Gal selectivity ratio that was decreased relative the wild type, but higher than for H450G [32]. We will hereafter refer to V546C, H450G and H450G/V546C as the GAL mutants.

In the case of Gal turnover by wild-type *Tm*P2O, we originally hypothesized that a possible reason for the preference of *Tm*P2O for Glc over Gal may be the limited space for accommodating the axial C4 hydroxyl at the *re* side of the flavin isoalloxazine ring [16], [31], [34]. Part of this spatial restriction would be due to the presence of the Thr169 side chain, which is positioned near the flavin N(5)/O(4) locus. Thr169 performs crucial functions in the catalytic mechanism of *Tm*P2O, such as regulating the formation of the transient flavin C(4a)-peroxide intermediate during the oxidative half-reaction with O_2_ as electron acceptor [35], ensuring proper protein flavinylation [31], and acting as a conformational switch between the reductive and oxidative half-reactions [19].

In this work, we set out to analyze principal binding modes of Glc and Gal in selected variants from previous site-saturation mutagenesis experiments that displayed more equal efficiencies in oxidation of Glc and Gal. Specifically, variants V546C, H450G and H450G/V546C were analyzed structurally, as well as H167A that served as a wild-type control. The overall aim with the study was to pinpoint binding modes that could help explain the altered specificity pattern, and provide guidelines for future engineering of *Tm*P2O variants for use in lactose conversion. A total of 16 high-resolution crystal structures of the *Tm*P2O mutants in complex with 2- or 3-fluorinated Glc and Gal are described, of which two structures have been reported previously (2IGO [14] and 3PL8 [19]). Based on these results, structural determinants for regioselective and differential oxidation of Glc and Gal are discussed. Extending from this, we attempt to rationalize the shift in relative preference between Glc to Gal for these *Tm*P2O GAL variants.

## Results

**Figure 1:**
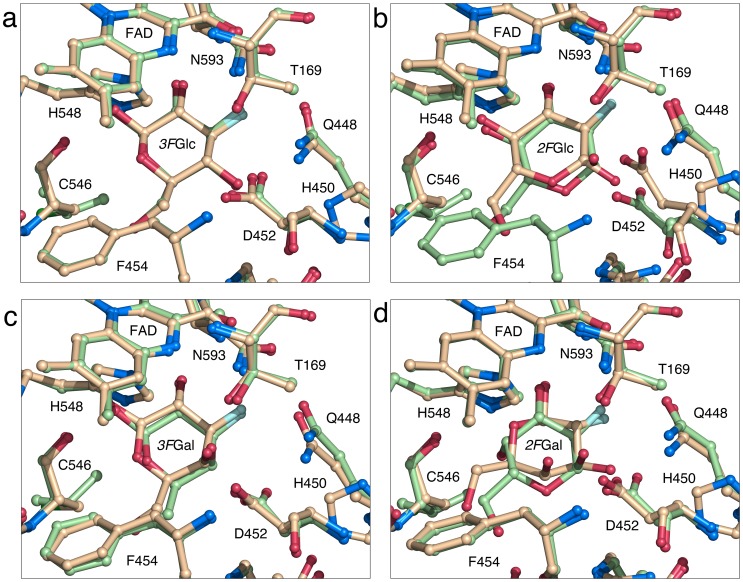
Principal productive and competing binding modes for fluorinated glucose and galactose. Structural overlay of the active sites in *Tm*P2O V546C (beige) and H450G (green). The V546C mutant is used as a reference since it displays the same binding modes as the H167A mutant, and for 2- or 3-fluorinated glucose, also agrees with the binding modes observed for the wild type. (a) Binding of *3F*Glc in the productive 2-oxidation binding mode. The sugar is stabilized as the *β*-anomer with O2 coordinated by His458 and Asn593 and C2 appropriately positioned for oxidation. The substrate-binding loop is in the semi-open conformation positioning Phe454 closely packed against the pyranose as has been described for the productive binding mode earlier [19]. (b) *2F*Glc in the competing 3-oxidation binding mode. In H450G, the C1 hydroxyl in *2F*Glc is stabilized in axial configuration (*α*-anomer) by Asp452 and Thr169 and the substrate-binding loop assumes the semi-open conformation. V546C stabilizes the *β*-anomer and reveals the open conformation of the substrate-binding loop as observed earlier for H167A in complex with *2F*Glc [14]. (c) *3F*Gal in productive 2-oxidation binding mode with the axial C4 hydroxyl group coordinated by Asp452 and Thr169. The substrate-binding loop is in the semi-open conformation. (d) *2F*Gal in competing binding modes. The competing binding mode observed for V546C corresponds to the *2F*Gal *β*-anomer oriented for oxidation at C1. The competing binding mode for H450G shows the *α-*anomer of *2F*Gal oriented for oxidation at C3. In both cases, the substrate-binding loop assumes the semi-open conformation compatible with the productive sugar-oxidation mode. All structures that bind sugar substrate show the Thr169 Oγ1 atom pointing *away* from the flavin N(5)/O(4) locus, which constitutes an additional hallmark of the productive binding mode. For clarity, the covalent link between the flavin and His167 is not shown in the pictures. The pictures were produced using the program PyMOL [43].

**Figure 2:**
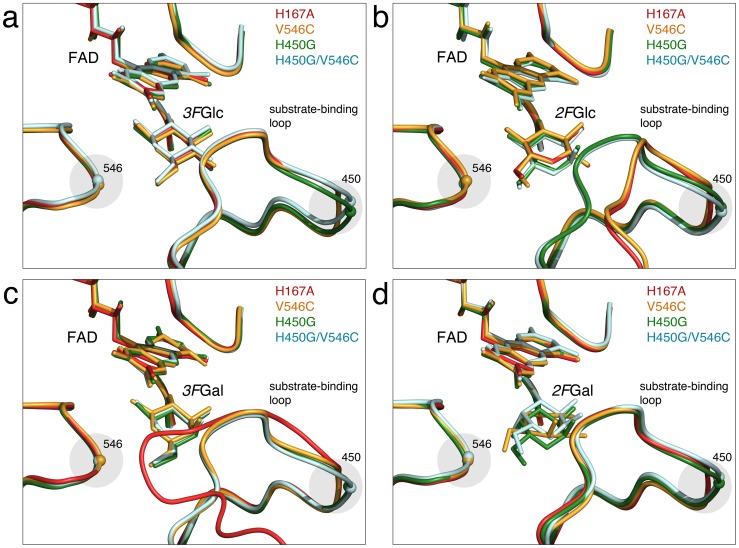
Comparison of conformation of the substrate-binding loop in *Tm*P2O complexes with fluorinated sugars. Superposition of mutant structures emphasizing the conformation of the substrate-binding loop. The FAD molecule and the pyranose sugar are shown as ball-and-stick models. (a) *Tm*P2O variants complexed with *3F*Glc corresponding to the productive 2-oxidation binding mode with the substrate-binding loop in the semi-open conformation. The relaxation induced by the H450G replacement (green and light-blue models) is highlighted by a shaded circle. The H167A model corresponds to PDB code 3PL8 [19]. (b) Mutant complexes with bound *2F*Glc in the competing 3-oxidation binding mode. The H167A (PDB code 2IGO [14]) and V546C variants show the open loop conformation, which also has been observed for the wild type. H450G and H450G/V546C show the productive semi-open loop conformation. (c) Mutant complexes of V546C, H450G and H450G/V546C with bound *3F*Gal in the productive 2-oxidation binding mode with the substrate-binding loop in the semi-open conformation. The wild-type mimic H167A displays did not bind the sugar and displays the closed, occluded loop conformation that is typically observed for *Tm*P2O in the absence of oxidizable sugar. The closed loop conformation is incompatible with sugar binding. (d) Mutant complexes with bound *2F*Gal. Despite the fundamentally different competing modes observed for H167A and V546C (C1-oxdiation mode) and H450G and H450G/V546C (C3-oxidation mode), all complexes show the substrate-binding loop in the semi-open loop conformation associated with productive sugar binding. The pictures were produced using the program PyMOL [43].

Data collection and refinement statistics for all data sets are given in Table 1 (Glc complexes) and Table 2 (Gal complexes), and unbiased electron-density maps are shown for all ligand complexes in Figure S1 (Glc complexes) and S2 (Gal complexes). The full description of protein-sugar interactions for the analyzed crystal complexes is given in Table 3 (Glc complexes) and Table 4 (Gal complexes). An illustrated overview of principal binding modes are given in Figure 1 and Figure 2, whereas detailed ligand-binding information is provided graphically in Figure S3 (Glc complexes) and Figure S4 (Gal complexes). The most important structural features are discussed below.

**Table 1:** Data collection and crystallographic refinement statistics for mutant P2O structures with fluorinated glucoses.

Data collection^1^						
Protein variant	H450G_3FGlc_	H450G_2FGlc_	V546C_3FGlc_	V546C_2FGlc_	H450G/V546C_3FGlc_	H450G/V546C_2FGlc_
Cell constants a, b, c (Å); β (°)	100.068, 102.742, 137.692; 90.80	102.340, 102.340, 128.510	102.507, 102.507, 128.626	102.490, 102.490, 118.101	101.504, 101.504, 238.672	100.079, 102.520, 137.339; 90.92
Space group/molecules per a.s.u.	P2_1_/4	P4_2_2_1_2/1	P4_2_2_1_2/1	P4_2_2_1_2/1	P4_1_2_1_2/2	P2_1_/4
Beamline, λ (Å)	MAX II, I911-2, 1.0379	MAX II, I911-3, 0.9600	PETRA III, P13,0.9465	MAX II, I911-3, 1.0000	PETRA III, P13, 0.9465	MAX II, I911-2, 1.0379
Resolution range, all (Å)	57.19–1.90	54.42–1.85	48.11–2.00	51.24–2.10	47.73–1.90	57.05–2.00
Resolution range, outer shell (Å)	2.00–1.90	1.90–1.85	2.10–2.00	2.20–2.10	2.00–1.90	2.10–2.00
Unique reflections	214,367 (30,620)	58,446 (4,176)	46,860 (6,246)	37,326 (4,775)	98,867 (13,879)	186,784 (25,332)
Multiplicity	3.2 (3.2)	14.3 (11.9)	26.5 (24.9)	14.4 (14.7)	10.9 (10.3)	3.6 (3.6)
Completeness (%)	97.8 (98.4)	99.4 (93.8)	99.8 (99.3)	99.9 (100)	100 (100)	99.8 (99.7)
<I/σI>	12.4 (2.5)	17.4 (3.2)	9.8 (3.1)	17.4 (4.8)	10.8 (2.0)	12.0 (2.2)
R_sym_ 2 (%)	11.6 (85.1)	13.4 (86.8)	30.8 (147.1)	14.2 (71.1)	17.8 (139.3)	10.4 (67.7)
CC(1/2)^3^	99.3 (56.3)	99.9 (86.8)	99.6 (80.8)	99.9 (92.4)	99.7 (61.1)	99.5 (70.9)
**Crystallographic refinement**						
Resolution range, all (Å)	50–2.00	50–1.85	48–1.85	40–2.10	45.88–1.90	50–2.00
Resolution range, outer shell (Å)	2.10–2.00	1.95–1.85	2.11–2.00	2.21–2.10	2.00–1.90	2.11–2.00
Completeness, all % (outer bin)	97.7 (98.9)	99.4 (96.3)	99.8 (99.3)	99.9 (100)	100 (100)	99.8 (99.7)
R_factor_ 4/work reflns, all	0.196/180,555	0.159/56,649	0.186/45,898	0.164/36,162	0.196/96,838	0.173/183,034
R_free_/free reflns, all	0.242/3,290	0.194/1,796	0.224/962	0.221/1,163	0.245/2,000	0.222/3,748
Number of amino-acid residues	2,301	573	573	576	1,152	2,301
Non-hydrogen atoms	19,624	5,199	4,993	4,956	9,835	20,416
Mean B (Å^2^) protein all/mc/sc	25.9/24.8/27.0	19.6/18.3/21.0	24.8/23.9/25.8	27.6/26.5/28.7	26.7/25.7/27.8	29.3/28.2/30.5
Mean B (Å^2^) solvent/N^o^. mol.	26.5/1,116	29.1/622	30.1/411	34.4/338	28.7/635	35.0/1,926
Rmsd bond lengths (Å), angles (°)	0.020, 2.01	0.022, 1.98	0.019, 1.89	0.018, 1.89	0.019/2.03	0.019, 1.99
Ramachandran^5^: favored (%)/allowed (%)/Outliers	97.4/99.9/2	98.1/100/0	96.8/100/0	97.2/99.8/1	97.3/99.7/3	97.1/99.9/2
PDB accession code	4MOE	4MOF	4MOG	4MOH	4MOI	4MOJ

1 The outer shell statistics of the reflections are given in parentheses. Shells were selected as defined in XDS [36] by the user.

2 R_sym_ = [Σ_hkl_ Σ_i_ |I–<I>|/Σ_hkl_ Σ_i_ |I| ]×100%.

3 CC(1/2) = Percentage of correlation between intensities from random half-datasets. Values given represent correlations significant at the 0.1% level [44].

4 R_factor_ = Σ_hkl_ | |F_o_|–|F_c_| |/Σ_hkl_ |F_o_|.

5 As determined by MolProbity [45].

**Table 2:** Data collection and crystallographic refinement statistics for mutant P2O structures with fluorinated galactoses.

Data collection^1^								
Protein variant	H167A_3Fgal_	H167A_2Fgal_	H450G_3Fgal_	H450G_2Fgal_	V546C_3Fgal_	V546C_2Fgal_	H450G/V546C_3Fgal_	H450G/V546C_2Fgal_
Cell constants a, b, c (Å); β (°)	99.875, 102.295, 136.957; 90.78	100.291, 102.476, 137.423; 91.02	99.896, 102.423, 137.365; 91.19	101.672, 101.672, 127.562	99.971, 102.980, 137.654; 90.92	101.724, 101.724, 251.284	100.242, 102.469, 137.840; 91.09	102.423, 102.423, 119.033
Space group/molecules per a.s.u.	P2_1_/4	P2_1_/4	P2_1_/4	P4_2_2_1_2/1	P2_1_/4	P4_3_2_1_2/2	P2_1_/4	P4_2_2_1_2/1
Beamline, λ (Å)	Diamond, I24,0.9191	Diamond, I24,0.9191	MAX II, I911-3,1.0000	MAX II, I911-3,1.0000	Diamond, I24,0.9191	SLS PX1 (X06SA),1.0000	PETRA III, P13,0.9465	Diamond, I24,0.9191
Resolution range, all (Å)	47.92–1.90	48.01–2.00	57.14–1.90	54.03–1.65	48.23–2.30	47.32–1.60	49.33–1.50	47.04–1.80
Resolution range, outer shell (Å)	2.00–1.90	2.10–2.00	2.00–1.90	1.70–1.65	2.40–2.30	1.70–1.60	1.60–1.50	1.90–1.80
Unique reflections	216,233 (30,699)	184,127 (24,662)	215,840 (30,624)	80,572 (6,649)	123,398 (14,694)	173,572 (28,468)	442,699 (77,654)	59,195 (8,727)
Multiplicity	6.7 (6.8)	6.9 (7.0)	3.7 (3.7)	12.9 (9.0)	6.1 (6.2)	25.3 (25.6)	7.4 (7.3)	26.1 (27.1)
Completeness (%)	99.8 (99.8)	98.2 (97.2)	99.2 (99.1)	99.7 (97.6)	99.5 (99.6)	100 (100)	99.7 (99.7)	100 (100)
<I/σI>	13.2 (2.1)	11.5 (2.4)	10.3 (2.6)	27.1 (3.8)	8.7 (1.6)	14.1 (2.2)	11.9 (1.6)	15.4 (2.1)
R_sym_ 2 (%)	10.8 (102.0)	17.6 (93.8)	20.6 (88.7)	7.4 (71.3)	17.2 (129.3)	23.1 (164.7)	9.2 (125.3)	26.0 (236.4)
CC(1/2)^3^	99.8 (75.1)	99.4 (75.8)	98.3 (73.5)	99.9 (81.7)	99.4 (65.8)	99.8 (73.4)	99.9 (59.0)	99.8 (75.1)
**Crystallographic refinement**								
Resolution range, all (Å)	47.92–1.90	48.01–2.00	50–1.90	50.0–1.65	48.23–2.30	47.32–1.60	49.33–1.50	47.04–1.80
Resolution range, outer shell (Å)	2.00–1.90	2.11–2.00	2.00–1.90	1.74–1.65	2.36–2.30	1.69–1.60	1.58–1.50	1.90–1.80
Completeness, all % (outer bin)	99.8 (99.8)	98.2 (97.1)	99.2 (99.1)	99.8 (98.5)	99.5 (99.6)	100 (100)	99.7 (99.7)	100 (100)
R_factor_ 4/work reflns, all	0.170/215,167	0.186/182,281	0.239/211,975^5^	0.192/78,664	0.194/122,372	0.182/171,834	0.156/441,592	0.162/58,172
R_free_/free reflns, all	0.214/1,066	0.222/1,846	0.280/3,865^5^	0.224/1,907	0.248/984	0.212/1,737	0.198/1,107	0.199/1,022
Number of amino-acid residues	2,297	2,295	2,300	576	2,300	1,139	2,301	576
Non-hydrogen atoms	20,179	20,233	19,522	5,115	19,647	10,344	21,660	5,035
Mean B (Å^2^) protein all/mc/sc	32.5/31.1/33.9	34.4/32.8/36.1	24.7/23.6/25.9	17.6/16.7/18.6	29.6/38.6/40.6	17.6/16.3/19.0	24.4/22.9/25.9	22.4/21.0/23.8
Mean B (Å^2^) solvent/N^o^. mol.	35.5/1,793	40.2/1,766	26.1/1,067	25.3/514	34.2/1,174	26.0/1,135	35.5/3,062	30.1/405
Rmsd bond lengths (Å), angles (°)	0.019, 1.90	0.019, 1.92	0.018, 1.96	0.021, 2.11	0.015, 1.76	0.023, 2.16	0.027, 2.28	0.020, 2.06
Ramachandran^6^: favored (%)/allowed (%)/Outliers	97.0/99.9/2	97.2/100/0	96.9/100/1	97.4/100/0	97.1/100/1	97.8/100/0	97.8/100/0	97.9/100/0
PDB accession code	4MOK	4MOL	4MOM	4MOO	4MOP	4MOQ	4MOR	4MOS

1 The outer shell statistics of the reflections are given in parentheses. Shells were selected as defined in XDS [36] by the user.

2 R_sym_ = [Σ_hkl_ Σ_i_ |I–<I>|/Σ_hkl_ Σ_i_ |I| ]×100%.

3 CC(1/2) = Percentage of correlation between intensities from random half-datasets. Values given represent correlations significant at the 0.1% level [44].

4 R_factor_ = Σ_hkl_ | |F_o_|–|F_c_| |/Σ_hkl_ |F_o_|.

5 We note that the R and R_free_ values are suspiciously high a 1.9-Å resolution model with electron density of good quality. Data sanity tests excluded twinning, pseudotranslation, and misindexing as possible reasons. However, the data suffer from poor completeness in the low-resolution region and contain several ice rings, which may account for the problems encountered during refinement.

6 As determined by MolProbity [45].

**Table 3:** Details of oxidation-binding modes for 2- or 3-fluorinated glucose.

	Glucose															
	Productive 2-oxidation binding mode^a^								Competing binding mode							
Variant	H167A^b^		V546C		H450G		H450G/V546C		H167A^c^		V546C		H450G		H450G/V546C	
Ligand	3FGlc		3FGlc		3FGlc		3FGlc		2FGlc		2FGlc		2FGlc		2FGlc	
Oxidation site	C2		C2		C2		C2		C3		C3		C3		C3	
Stereoisomer	β		β		β		β		β		β		α^d^		α^d^	
Loop conformer	semi-open		semi-open		semi-open		semi-open		open		open		semi-open		semi-open	
Sugar-protein interactions e	O1	V546 O	O1	V546 O	O1	V546 O	O1	V546 O	O1	Q448 Nε2	O1	Q448 Nε2	O1	D452 Oδ2	O1	D452 Oδ2
	–	–	–	–	O1	H548 Nε2	O1	H548 Nε2	–	–	–	–	O1	T169 Oγ1	O1	T169 Oγ1
	O2	H548 Nε2	O2	H548 Nε2	O2	H548 Nε2	O2	H548 Nε2	F2	Q448 Nε2	F2	Q448 Nε2	F2	Q448 Nε2	F2	Q448 Nε2
	O2	N593 Nδ2	O2	N593 Nδ2	O2	N593 Nδ2	O2	N593 Nδ2	O3	H548 Nε2	O3	H548 Nε2	O3	H548 Nε2	O3	H548 Nε2
	F3	Q448 Nε2	F3	Q448 Nε2	F3	Q448 Nε2	F3	Q448 Nε2	O3	N593 Nδ2	O3	N593 Nδ2	O3	N593 Nδ2	O3	N593 Nδ2
	O4	D452 Oδ2	O4	D452 Oδ2	O4	D452 Oδ2	O4	D452 Oδ2	O4	V546 O	O4	V546 O	O4	V546 O	O4	V546 O
	O6	Y456 Oη	O6	Y456 Oη	O6	Y456 Oη	O6	Y456 Oη	–	–	–	–	O6	Y456 Oη	O6	Y456 Oη

a The following three criteria are considered consistent with a productive binding mode: (i) The sugar is oriented for oxidation at C2; (ii) the substrate-binding loop is in the semi-open conformation; (iii) the side chain Oγ1 group of Thr169 is pointing away from the flavin N(5)/O(4) locus.

b PDB code 3PL8 [19].

c PDB code 2IGO [14].

d The ability of P2O variants to stabilize either the α- or β-anomer is uncorrelated to space group and crystal contacts, and depends solely on the new structural context provided by the mutation.

e Italicized interactions represent interactions with the catalytic residues.

**Table 4:** Details of oxidation-binding modes for 2- or 3-fluorinated galactose.

	Galactose															
	Productive 2-oxidation binding mode a								Competing binding mode							
Variant	H167A		V546C		H450G		H450G/V546C		H167A		V546C		H450G		H450G/V546C	
Ligand	3FGal		3FGal		3FGal		3FGal		2FGal		2FGal		2FGal		2FGal	
Oxidation site	–		C2		C2		C2		C1		C1		C3		C3	
Stereoisomer	–		β		β		β		β		β		α^b^		α^b^	
Loop conformer	closed		semi-open		semi-open		semi-open		semi-open		semi-open		semi-open		semi-open	
Sugar-protein interactions c	–	–	O1	V546 O	O1	V546 O	O1	V546 O	O1	H548 Nε2	O1	H548 Nε2	O1	D452 Oδ2	O1	D452 Oδ2
	–	–	–	–	O1	H548 Nε2	–	–	O1	N593 Nδ2	O1	N593 Nδ2	O1	T169 Oγ1	O1	T169 Oγ1
	–	–	O2	H548 Nε2	O2	H548 Nε2	O2	H548 Nε2	F2	Q448 Nε2	F2	Q448 Nε2	F2	Q448 Nε2	F2	Q448 Nε2
	–	–	O2	N593 Nδ2	O2	N593 Nδ2	O2	N593 Nδ2	F2	N593 Nδ2	F2	N593 Nδ2	–	–	F2	N593 Nδ2
	–	–	F3	Q448 Nε2	F3	Q448 Nε2	F3	Q448 Nε2	O3	D452 Oδ2	O3	D452 Oδ2	O3	H548 Nε2	O3	H548 Nε2
	–	–	–	–	F3	N593 Nδ2	–	–	–	–	O3	Q448 Nε2	O3	N593 Nδ2	O3	N593 Nδ2
	–	–	O4	D452 Oδ2	O4	D452 Oδ2	O4	D452 Oδ2	O4	D452 Oδ2	O4	D452 Oδ2	O4	V546 O	O4	V546 O
	–	–	O4	T169 Oγ1	O4	T169 Oγ1	O4	T169 Oγ1	O6	L545 O	O6	L545 O	–	–	–	–
	–	–	O6	Y456 Oη	O6	Y456 Oη	O6	Y456 Oη	O6	Y456 Oη	O6	Y456 Oη	O6	Y456 Oη	O6	Y456 Oη

a The following three criteria are considered consistent with a productive binding mode: (i) The sugar is oriented for oxidation at C2; (ii) the substrate-binding loop is in the semi-open conformation; (iii) the side chain Oγ1 group of Thr169 is pointing away from the flavin N(5)/O(4) locus.

b The ability of P2O variants to stabilize either the α- or β-anomer is uncorrelated to space group and crystal contacts, and depends solely on the new structural context provided by the mutation.

c Italicized interactions represent interactions with the catalytic residues.

### Structural Basis for Binding of 2- or 3-Fluorinated Glc and Gal by Variant H167A (Wild-Type Mimic)

#### Analysis of binding modes of Glc

We reported previously the wild-type mimic H167A in complex with the *β*-anomer of *3F*Glc (PDB code 3PL8 [19]), bound in the productive binding mode for C2 oxidation (Table 3; Fig. S3 *a*). Based on the observed binding, three principal criteria can be defined as characteristic structural features of a productive C2-oxidation-competent binding mode in the *Tm*P2O active site during the reductive half-reaction: *i.e.*, (1) the sugar is bound in the *β*-anomeric form oriented for oxidation at C2 (identical to V546C in Fig. 1*a*), placing the equatorially configured O2 hydroxyl within favorable hydrogen-bonding distance of His548 Nε2 and Asn593 Nδ2; (2) the active-site loop is well-ordered in a semi-open conformation (Fig. 2*a*); and (3) the Oγ1 atom of Thr169 is pointing away from the flavin N(5)/O(4) locus [14], [19].

When the competing Glc-binding mode is concerned, *i.e*., H167A–*2F*Glc (PDB code 2IGO [14]), *2F*Glc is bound as the *β*-anomer in position for 3-oxidation (Table 3; identical to V546C in Fig. 1*b*; Fig. S3 *b*), and the substrate-recognition loop adopts an open conformer with less favorable active-site packing (Fig. 2*b*).

#### Analysis of binding modes of Gal

In the complex of H167A with 3-deoxy-3-fluoro-D-galactose, H167A–*3F*Gal (emulating a competent 2-oxidation mode of Gal), no ligand is observed in the active site (Table 4; Fig. S4 *a*) and the substrate-recognition loop assumes the fully closed conformation (Fig. 2*c*), which is incompatible with sugar binding during the reductive half-reaction, a structural state that has been previously observed in *Tm*P2O structures without bound sugar (PDB code 1TT0 [12]). Similar to sugar-free *Tm*P2O structures, the H167A–*3F*Gal complex has the Thr169 side chain hydrogen bonding to the flavin N(5)/O(4) locus. Clearly the 2-oxidation binding mode of Gal must be possible for H167A since Gal is turned over by *Tm*P2O to produce the 2-keto product. We do not suggest that the absence of *3F*Gal in H167A implies that the sugar is unable to bind, but rather that it indicates that this binding mode is too unfavorable to be stably trapped in a crystal complex.

In the complex with H167A and 2-deoxy-2-fluoro-D-galactose (H167A–*2F*Gal), the sugar is unexpectedly neither bound in the 2-oxidation or 3-oxidation mode, but in an orientation where the Gal *β*-anomer is positioned for oxidation at C1 (Table 4; Fig. S4 *b*) indicating that the 1-oxidation binding mode is a more relevant competing, or inhibiting, state when Gal is the substrate. As expected for a competent sugar-bound state, the Thr169 side chain is pointing away from the flavin N(5)/O(4) locus, and the substrate-recognition loop is in the semi-open conformation (Fig. 2*d*).

### Structural Basis for Binding of 2- or 3-Fluorinated Glc and Gal by Variant V546C

#### Analysis of binding modes of Glc

Based on the overall more similar kinetics with Glc and Gal, we expected the V546C mutant to behave structurally more similar to H167A (and the wild type) than does the H450G variant. As observed for the wild-type mimic H167A–*3F*Glc [19], the V546C–*3F*Glc complex shows that the active site stabilizes the *β*-anomeric form of Glc oriented for 2-oxidation (Table 3; Fig. 1*a*; Fig. S3 *c*). Thr169 Oγ1 is pointing away from the flavin, and the substrate-recognition loop is present in the semi-open state (Fig. 2*a*). The differences between the wild-type mimic H167A and V546C when binding 3FGlc are very subtle. Upon replacement, the second branching methyl group in Val546 is eliminated, allowing the introduced cysteine to relax the 546 backbone, which in turn allows the Cβ atom of residue 546 to move slightly closer to the sugar.

Also the competing 3-oxidation mode represented by V546C–*2F*Glc is identical to that of H167A–*2F*Glc with the *β*-anomer oriented for oxidation at C3 (Table 3; Fig. 1*b*; Fig. S3 *d*). The substrate-recognition loop adopts the fully open conformation (Fig. 2*b*), and Thr169 is pointing away from the flavin. Thus, for the competing 2- and 3-oxidation binding modes of Glc, the V546C mutant is indeed structurally almost identical to *Tm*P2O H167A [14], [19] and the wild type (unpublished results).

#### Analysis of binding modes of Gal

Unlike H167A where no binding of *3F*Gal was observed, V546C stabilizes the *β*-anomer of *3F*Gal in the expected 2-oxidation mode (Table 4; Fig. 1*c*; Fig. S4 *c*). The loop is in the semi-open state (Fig. 2*c*), and Thr169 is pointing away from the flavin. Thus, the binding of Gal is identical to that of Glc binding for productive oxidation at C2.

For the competing binding mode, represented by the V546C–*2F*Gal complex, the structural features are identical to that of H167A: *2F*Gal bound for oxidation at C1 (Table 4; Fig. 1*d*; Fig. S4 *d*), stabilized in the *β*-anomeric configuration showing the substrate-recognition loop in the semi-open conformation (Fig. 2*d*), and the Thr169 side chain hydroxyl group pointing away from the flavin N(5)/O(4) locus.

### Structural Basis for Binding of 2- or 3-Fluorinated Glc and Gal by Variant H450G

#### Analysis of binding modes of Glc

The variant H450G binds *3F*Glc in the productive Glc 2-oxidation mode (Table 3; Figure 1*a*; Fig. S3 *e*) identical to that of *Tm*P2O H167A (PDB code 3PL8 [19]), the wild type (unpublished results) and V546C (above). Compared with H167A and V546C, which retain His450, the backbone at position 450 in H450G is relaxed by the His450→Gly replacement, beyond which only minor structural changes in the overall active site accompany the mutation. The substrate-recognition loop in the semi-open conformation (Fig. 2*a*), and the Thr169 side chain hydroxyl group pointing away from the flavin N(5)/O(4) locus.

Interestingly, the competing binding mode represented by *2F*Glc is stabilized by the H450G active site in its *α*-anomeric state (Table 3; Figure 1*b*; Fig. S3 *f*). The axial O1 of the *2F*Glc *α*-anomer is stabilized specifically by Asp452 Oδ2 and Thr169 Oγ1. Besides the axial configuration of the 1-hydroxyl group and an additional hydrogen bond to Tyr456, H450G forms a similar set of interactions with *2F*Glc as do H167A and V546C. For both H450G–*3F*Glc and H450G–*2F*Glc, the substrate-recognition loop is in the productive semi-open conformation (Fig. 2*b*), and Thr169 Oγ1 points away from the flavin.

#### Analysis of binding modes of Gal

In the H450G complex with *3F*Gal, the sugar is present as the *β*-anomer bound in the expected 2-oxidation mode (Table 4; Fig. 1*c*; Fig. S4 *e*). The substrate-recognition loop is in the semi-open state (Fig. 2*c*), and Thr169 Oγ1 points away from the flavin. Notably, the axial C4 hydroxyl in the *β*-anomer of *3F*Gal bound in the 2-oxidation-binding mode assumes the same position as the axial O1 in the *α*-anomer of *2F*Glc (Glc in orientation for 3-oxidation), receiving stabilizing interactions from Asp452 Oδ2 and Thr169 Oγ1.

As expected when fluorine is present at Gal C2, *2F*Gal is bound to H450G in the competing binding mode, *i.e.,* for oxidation at C3 (Table 4; Fig. 1*d*; Fig. S4 *f*). As observed for the H450G–*2F*Glc complex, *2F*Gal is present as the *α*-anomer with the axial C1 hydroxyl group stabilized by Asp452 Oδ2 and Thr169 Oγ1, but with an apparently strained conformation of the pyranose ring. The substrate-recognition loop is in the semi-open conformation (Fig. 2*d*), and Thr169 Oγ1 points away from the flavin.

### Structural Basis for Binding of 2- or 3-Fluorinated Glc and Gal by Variant H450G/V546C

The H450G/V546C variant combines the two mutations H450G and V546C, which individually promotes Gal turnover. For the Glc complexes (*3F*Glc and *2F*Glc), the H450G/V546C variant shows identical binding to that of H450G (Table 3; Figs. S3 *g* and S3*h*). Besides some minor differences originating from the V546C replacement, the same is true for the complexes with fluorinated Gal (*3F*Gal and *2F*gal; Table 4; Figs. S4 *g* and S4*h*). That H450G and H450G/V546C show identical stabilization of the *α*-anomeric configuration of *2F*Glc and *2F*Gal (3-oxidation mode of Glc and Gal), while V546C does not, supports that this is a unique property of introducing the His450→Gly replacement, and independent of the mutation at position 546.

## Discussion

The binding promiscuity of the *Tm*P2O active site allows a range of monosaccharides to be bound in a variety of binding modes and affinities. Although regioselectivity of at least 2-oxidation of Glc and Gal appears to be strictly regulated by the enzyme [19], [28], competing binding modes will inevitably exist, albeit only one productive binding mode will result in the desired catalytic outcome. By deliberately shifting the equilibrium of substrate-bound conformers towards different binding modes, structural details regarding alternative, and potentially relevant, binding states can be obtained. Detailed structural information regarding such binding modes is invaluable for creating more refined enzyme designs.

To get a better understanding of the nature of alternative binding modes and to help rationalize changes in substrate preference and conversion by engineered *Tm*P2O variants, our approach is to take advantage of the property of the *Tm*P2O active site to avoid a fluorine in the oxidation site, which is located at the *re* face the flavin N(5) atom and roughly midway between His548 Nε2 and Asn593 Nδ1. By using Glc fluorinated at C3 (*3F*Glc), the competing 3-oxidation binding mode is disabled, and a shift occurs towards the more favorable binding mode where Glc is oriented for productive 2-oxidation (PDB code 3PL8 [19]). Similarly, by using 2-fluorinated Glc (*2F*Glc), the enzyme-substrate conformer can be shifted towards the 3-oxidation binding mode, which provided structural descriptors for the principal competing binding mode (PDB code 2IGO [14]). Thus, this approach allows us to analyze and evaluate the relative structural stabilization of individual binding modes, and to use this information to aid rationalization of the structural determinants behind the observed kinetics, and possibly improve future enzyme designs.

We started this study with the initial assumption that, similar to what is observed for the oxidation of Glc [14], [19], catalysis of the productive 2-oxidation reaction of Gal may be in competition with a 3-oxidation binding mode. This was a rational initial assumption since catalysis of 2-oxidation of Glc or Gal by the wild type and H167A (wild-type mimic) displays similar kinetics [26], [28]. Therefore, to evaluate and compare the productive and competing sugar-binding modes for oxidation of Glc and Gal, we used crystal-structure analysis of 2- and 3-fluorinated Glc and Gal derivatives bound to the *Tm*P2O variants H167A, V546C, H450G and H450G/V546C.

Wild-type *Tm*P2O displays a clear preference for Glc over Gal. This is shown by a selectivity ratio of 177 in favor of Glc when O_2_ (air) is used as electron acceptor (Table 5), *i.e*., the ratio of the specificity constants for the two substrates, [(*k*_cat_/*K*_m_)_Glc_/(*k*_cat_/*K*_m_)_Gal_] [32]. The corresponding selectivity ratio for H450G is 9 in favor of Glc, meaning that if to achieve a more equal conversion efficiency of the two substrates, the relative preference for Glc over Gal is more favorable for H450G. For the V546C variant, a selectivity ratio of 210 shows that the relative efficiency is even less favorable than for the wild type. In the H450G/V546C mutant, the selectivity ratio with O_2_ as electron acceptor shows a 27-fold preference of Glc over Gal. Thus, all else being equal, the H450G mutant is the preferred variant for achieving a more balanced yield of products from Glc and Gal oxidation. Variants containing the T169G replacement show an even more reduced selectivity for these two sugars (selectivity ratios of 1.1 to 3.5, Table 3), this, however, comes at the expense of considerable decreases in *k*_cat_ by at least one order of magnitude.

**Table 5:** Apparent steady-state kinetic constants of TmP2O wild-type and mutants with D-glucose or D-galactose as electron donor, and O_2_ (air) under saturation as electron acceptor.

Enzyme variant	D-glucose/O_2_			D-galactose/O_2_			Substrate selectivity	Citation
	K_M_ (mM)	k_cat_ (s^–1^)	k_cat_/K_M_(mM^–1^⋅s^–1^)	K_M_ (mM)	k_cat_ (s^–1^)	k_cat_/K_M_(mM^–1^⋅s^–1^)	(k_cat_/K_M_)_Glc_/(k_cat_/K_M_)_Gal_	
Wild type	0.939±0.04	48.1±0.50	51.2	8.79±0.54	2.51±0.05	0.286	177	[32]
H167A^a^	5.97±0.37	2.06±0.03	0.35	n.d.	n.d.	n.d.	n.d.	
H450G	0.987±0.05	12.5±0.15	12.7	2.45±0.12	3.51±0.04	1.43	9	[32]
V546C	3.06±0.14	88.6±1.30	29.0	46.2±3.21	6.57±0.15	0.142	210	[33]
H450G/V546C	2.43±0.14	16.8±0.27	13.5	12.0±0.38	5.92±0.05	0.493	27	[32]
T169G	0.69±0.11	0.26±0.01	0.38	2.48±0.94	0.27±0.02	0.11	3.5	[34]
T169G/V546C	0.44±0.02	0.43±0.01	0.99	0.40±0.09	0.38±0.01	0.94	1.1	[33]

a Since H167A was not designed to specifically improve galactose turnover, but to slow down flavin reduction during the reductive half-reaction, the kinetic constants for H167A were not determined. The mutant was included since it is a close structural mimic of the wild-type when sugar binding is concerned. Its purpose is therefore mainly for structural comparisons.

### Glucose Binding

Binding of *3F*Glc in the productive 2-oxidation binding mode does not differ significantly between the mutant enzymes at a structural level (Table 3; Figure 1*a*; Fig. S3 *a,c,e,g*). This suggests that the productive 2-oxidation binding mode of Glc is not *per se* affected by the mutations. In the H450G–*3F*Glc complex (mimic of the productive binding mode for Glc), the mutation is accompanied by only minor structural changes in the overall active site, including mainly relaxation of the backbone at position 450. This is consistent with an unperturbed *K*_m_[Glc] in H450G compared with the wild type (H450G, *K*_m_ 0.987 mM; wild type, *K*_m_ 0.939 mM; Table 5) [32]. However, turnover of Glc is decreased 3.8-fold (H450G, *k*_cat_ 12.5 s^−1^; wild type, *k*_cat_ 48.1 s^–1^; Table 5), indicating a certain amount of transition-state destabilization for the overall reaction. His450 is close to both Asp452 and Arg472 (important determinants of regioselective 2-oxidation of Glc) when the active-site loop is in the productive semi-open conformation [19].

When the competing binding mode (3-oxidation) is concerned, there is a clear difference between the more “wild-type-like” variants H167A and V546C compared with the variants carrying the H450G replacement. In H167A and V546C, *2F*Glc is present as the *β*-anomer (Table 3; Fig. 1*b*; Fig. S3 *b* and S3*d*), and the substrate-recognition loop is fully open (Fig. 2*b*). In H450G and H450G/V546C, however, *2F*Glc oriented for oxidation at C3 is stabilized in its *α*-anomeric configuration with a well-ordered semi-open substrate-recognition loop conformation (Fig. 2*b*); the *α*-anomeric O1 hydroxyl group is firmly coordinated by Asp452 Oδ2 and Thr169 Oγ1, and additional hydrogen bonds between the substrate and protein are possible (Table 3). This suggests that in mutants carrying the H450G replacement, the competing 3-oxidation binding mode for α-*2F*Glc, and possibly also for non-fluorinated Glc, receives a potentially larger degree of stabilization (as judged by the number of possible interactions; Table 3), than does *β*-anomeric *2F*Glc in H167A and V546C.

Since the structural integrity and conformation of Thr169 is crucial for proper wild-type catalytic activity, its involvement in coordinating the O1 hydroxyl group of *2F*Glc in the H450G variants is interesting. Conceivably, this interaction leads to unfavorable conformational restriction of the threonine side chain, forming an *α*-anomeric trap for Thr169 that stabilizes the competing 3-oxidation binding mode, and may prevent the substrate from rapid dissociation and re-binding in a productive mode. This is expected to result in an overall lower turnover rate of Glc 2-oxidation for H450G and H450G/V546C relative to H167A and V546C, which is also the case (Table 5). The importance of Thr169 is discussed in more detail below.

In the case of H167A, it has been suggested that the two binding pathways observed for this mutant may arise because of the increased flexibility of the non-covalently bound FAD and more spatial freedom below the flavin ring to accommodate additional binding modes of glucose [28]. As shown here, the existence of multiple binding modes for Glc and Gal in the active site of *Tm*P2O is not due to the H167A replacement and flavin ring flexibility, since V546C with intact flavinylation shows identical binding modes of *3F*Glc, *2F*Glc, *3F*Gal and *2F*Gal as does H167A.

### Galactose Binding

We speculated earlier that steric hindrance at the *re*-face of the flavin isoalloxazine ring might interfere with binding of the axial C4 hydroxyl in Gal, and at least partly explain the low turnover rates of Gal by *Tm*P2O [16], [31], [34]. Although this is partly true, it is not obvious that any one single side chain would account for the steric interference. Rather, based on the data obtained in this work and discussed further below, it appears to be an overall steric effect at several positions. The steric hindrance is effectively relieved by subtle relaxation at key backbone positions in the active site, either 546 or 450, or both, which allows for more favorable binding modes of Gal.

The wild-type mimic, H167A, shows an impaired ability to bind *3F*Gal productively for C2 oxidation. We base this statement on the absence of a bound sugar in the H167A–*3F*Gal crystal structure (Fig. S4 *a*), and that the active-site conformer is in the fully occluded state (Fig. 2*c*), a state that is incompatible with sugar binding during the reductive half-reaction [12], [14], [19]. To confirm this observation, we collected multiple data sets from H167A–*3F*Gal crystals. Although the best ordered crystal included here corresponds to the H167A structure without bound sugar, two other data sets show evidence of partly occupied states of *3F*Gal that agree with the *3F*Gal-binding mode observed for the other variants (data not shown). In one of these data sets there is a nearly perfect 50∶50 composite electron-density map of the unbound occluded state and the 2-oxidation mode of *3F*Gal, and in another data set, the *3F*Gal-bound state is dominating with traces of the occluded state. For all the latter two cases, there are also varying relative ratios of the two states between different subunits in the homotetramer. Contrary to H167A, the *3F*Gal-bound states for the other mutants (Fig. S4 *c,e,g*) show more consistent binding and high agreement between subunits, indicating that *3F*Gal is more favorably bound. This corroborates that productive 2-oxidation binding of *3F*Gal (*i.e.*, ligand inducing the productive 2-oxidation mode), and likely also non-fluorinated Gal, is somehow unfavorable in H167A (and most likely also in the wild type), and that the ensemble of active-site conformers is shifted towards the closed state that excludes sugar substrates.

All other mutant *3F*Gal complexes reported here (V546C–*3F*Gal, H450G–*3F*Gal and H450/V546C–*3F*Gal) bind the productive 2-oxidation state of *β*-anomeric *3F*Gal with a similar set of protein-sugar interactions (Table 4; Figure 1*c*; Fig. S4 *c,e,g*). Stabilizing interactions are offered by Asp452 Oδ2 and Thr169 Oγ1 to the axial C4 hydroxyl group in Gal, which is expected to facilitate binding of Gal oriented for productive 2-oxidation, presumably also to promote conversion to the 2-keto galactose product. The only significant difference is that two additional hydrogen bonds are possible for H450G–*3F*Gal (Table 4). The correlation between structural data and the kinetics (Table 5) with O_2_ as electron acceptor is fairly consistent. All GAL mutants show higher turnover numbers for Gal with O_2_ as acceptor compared with the wild type, and H450G also shows a lower *K*_m_, which may reflect better binding due to the additional hydrogen bonds.

Analyses of the *2F*Gal complexes (Table 4) show that for the wild-type-like variants H167A and V546C, 2-oxidation of Gal may be in competition with a binding mode where the *β*-anomer is oriented for oxidation at C1 (Fig. 1*d*; Fig. S4 *b* and S4*d*). The C1 hydroxyl of *2F*Gal is exposed to the catalytic residues His548 and Asn593 in the “wrong” configuration for a productive catalytic event. Moreover, in V546C–*2F*Gal, the interaction of His548 with the β-configured O1 group of *2F*Gal pulls the O1 group and forces the pyranose ring towards a nearly planar configuration. As for the 3-oxidation binding mode of Glc, the H450G variants stabilize the 3-oxidation binding mode of Gal in the *α*-anomeric state, suggesting that this binding mode is a relevant alternative binding mode also for Gal. In H450G and H450G/V546C, the His450 side chain is absent, and similar steric hindrance does not exist. The backbone relaxation around position 450 (Fig. 2) provides the Asp452 backbone with increased positional freedom to adjust the carboxylate side chain for optimal interaction with the O4 group, and to stabilize the *α*-anomer of *2F*Gal in the 3-oxidation mode, which is therefore an intrinsic feature of the His450→Gly replacement (Fig. 1*d*; Fig. S4 *f* and S4*h*).

Why then do H167A and V546C stabilize *2F*Gal oriented for 1-oxidation instead of the 3-oxidation mode of *2F*Gal? A possible explanation could be that H167A and V546C are unable to stabilize the *α*-anomeric form of *2F*Gal. In H167A and V546C, His450 is forming two possible hydrogen bonds: His450 Nδ1– water and atom; and His450 Nε2– Arg472 Nη1; which positions the His450 Cε1 atom to face Asp452 Oδ2. For H167A or V546C to be able to stabilize the *α*-anomer of *2F*Gal, additional space is needed near the carboxyl group of Asp452 to accommodate the axial O1 of *2F*Gal. To achieve this, Asp452 would need to move closer to His450 bringing its Oδ2 atom within 2.8 Å of His450 Cε1, which is too close to be energetically acceptable. But even without the possibility of energetic penalty, it would still be problematic to allow proper readjustment of Asp452 without the relaxation offered by a glycine at position 450. While this could explain why H167A and V546C are unable to stabilize the *α*-anomer of *2F*Gal oriented for oxidation at C3, it does not explain why the *β*-anomer is not stabilized instead. Possibly, the answer lies in the tendency of these variants to distort Gal towards planar configuration at C1.

### Importance of Thr169

As discussed above, we suggested originally that the axial O4 group in Gal oriented for oxidation at C2 would experience steric hindrance in the vicinity of Thr169, and that this could be one of the reasons for the low efficiency of Gal turnover by wild-type *Tm*P2O [16], [34]. To test this hypothesis, a variant lacking a side chain at position 169, T169G, was investigated, and shown to have improved binding of Gal (3.5-fold decrease in *K*_m_[Gal], O_2_ as electron acceptor; Table 5). Although binding of Gal was improved, eliminating Thr169 proved to be detrimental to the catalytic activity of *Tm*P2O, causing a 10-fold lower *k*_cat_[Gal] compared with the wild type [35] (Table 5). Considering the importance of Thr169 for the oxidative half-reaction with oxygen [19], [31], [35], this result is not surprising.

For all GAL mutants investigated here, the axial O4 group in *3F*Gal can in fact be accommodated in the productive 2-oxidation binding mode without introducing a smaller side chain at position 169 (Table 4; Fig. S4). Not only is the axial O4 group spatially accommodated in the GAL mutants, but also receiving stabilizing hydrogen bonds from Asp452 and Thr169 (Table 4). Unlike T169G, all GAL mutants also show improved Gal turnover compared with the wild type (Table 5). In the case of the GAL mutants, subtle backbone changes in the active site provide conformational freedom of the active site to accommodate the axial O4 hydroxyl group without interfering with the function of Thr169. The most convincing example is the H450G variant where relaxation of the backbone allows fine-tuning of the substrate-binding protein side chains and accommodation of Gal. H450G has an identical *K*_m_[Gal] as T169G, but 1.4-fold higher *k*_cat_[Gal] compared with the wild type and 13-fold higher *k*_cat_[Gal] than T169G with O_2_ as electron acceptor. Thus, in an approach to improve the overall 2-oxidation of Gal by *Tm*P2O, mutagenesis should aim towards slight increases of the active-site volume, but as far as possible avoid changes at or close to position 169. For efficient catalysis, it is also of interest to consider which competing binding modes may be stabilized by the chosen mutation.

## Conclusions

An enzyme engineering effort to find a *Tm*P2O variant with improved Gal turnover, and more equal conversion rates for Glc and Gal, has identified the mutant H450G as an interesting candidate. This mutant displays a 3.8-fold lower *k*_cat_[Glc] with an unchanged *K*_m_[Glc], and a 1.4-fold higher *k*_cat_[Gal], and 3.6-fold lower *K*_m_[Gal] with O_2_ as electron acceptor [32]. Based on our crystal-structure analyses, one possible explanation for the lower turnover of Glc by H450G is an increased stabilization of the competing 3-oxidation binding mode of Glc through an *α*-anomeric trap involving Thr169. Since the enzyme needs to eject the substrate bound in this nonproductive binding mode in order to bind it productively, increased binding of the competing 3-oxidation binding mode will effectively slow down overall 2-oxidation turnover of Glc. Thus, the competing 3-oxidation binding mode would act as a dead-end enzyme-substrate complex that effectively slows down the catalytic cycle.

The higher Gal conversion rate may be attributed to a differential stabilization of the productive 2-oxidation binding mode through the possibility of additional hydrogen bonds and favorable geometry for 2-oxidation of Gal. Like Glc in the competing 3-oxidation binding mode, a similar *α*-anomeric trap exists in the 3-oxidation mode of Gal, however, compared with Glc, this binding mode seems less well stabilized by *Tm*P2O as judged by the apparent strain of the α-*2F*Gal molecules bound to H450G and H450G/V546C. We therefore do not expect the *α*-anomer of Gal to significantly influence the rate of establishing the productive 2-oxidation binding mode. The structural basis of the improved performance of H450G with Gal as substrate is rationalized by a relaxation of the backbone around position 450 governed by the His450→Gly mutation, which enables readjustment of a set of key substrate-binding residues to more optimally accommodate and bind the axial O4 group. These small but distinct changes work in concert to provide a more suitable framework for binding of Gal than what is possible in wild-type *Tm*P2O.

In this work, we have structurally defined a set of new substrate-binding modes for *Tm*P2O variants that may or may not compete with productive binding for 2-oxidation of D-glucose and D-galactose in the wild type. These binding modes appear well stabilized in the mutants investigated, and should be considered when interpreting Michaelis-Menten kinetics and when undertaking refined rational enzyme design. These results also provide useful information for the design and interpretation of results from future rapid kinetics experiments.

## Materials and Methods

### Crystallization and X-ray Data Collection

The mutagenesis, expression and biochemical characterization of the *Tm*P2O variants have been reported earlier: H167A [14], H450G [32], V546C [15], [33] and H450G/V546C [32]. Briefly, the mutant proteins were purified by immobilized metal ion affinity chromatography (IMAC) and buffer exchanged to 50 mM KH_2_PO_4_-buffer (pH 6.5). To prepare the proteins for crystallization, protein stock solutions at a concentration of 20 OD_280_/mL were prepared in 20 mM MES pH 5.2.

To avoid bias, all crystals were produced under identical conditions and soaked with fluorinated sugars according to the same standard procedure (see below). Crystals of *Tm*P2O variants H167A, H450G, V546C and H450G/V546C were grown using the hanging drop vapor diffusion method. Drops were prepared by equal volumes of protein stock solution and reservoir solution containing 0.1 M MES (pH 5.2), 50 mM MgCl_2_, and 10 (w/v)% monomethylether polyethylene glycol 2000 (mme PEG 2000). Under these conditions, *Tm*P2O crystallizes in a meta-stable packing mode that allows for minor adjustments leading to different space groups.

Ligand soaking and cryo protection were combined by transferring the crystals to a solution containing 0.1 M MES (pH 5.2), 50 mM MgCl_2_, and 10 (w/v)%, 28% mme PEG 2000, and dissolved ligand. Crystals were dipped in the solution and immediately plunged into liquid nitrogen for vitrification. The ligands used were: *3F*Glc (3-deoxy-3-fluoro-D-glucose; Toronto Research Chemicals Inc., Toronto, Canada; Cat. N^o^. D235000); *2F*Glc (2-deoxy-2-fluoro-D-glucose; Sigma-Aldrich Co. LLC., USA; Cat. N^o^. F5006-25 mg); *3F*Gal (3-deoxy-3-fluoro-D-galactose; Carbosynth Ltd., Newbury, UK; Cat. N^o^. MD05336); and *2F*Gal (2-deoxy-2-fluoro-D-galactose; Toronto Research Chemicals Inc.; Cat. N^o^. D233000).

A total of 14 data sets for *Tm*P2O variants H167A, V546C, H450G, and H450G/V546C complexed with *3F*Glc, *2F*Glc, *3F*Gal and *2F*Gal were collected using synchrotron radiation. We reported the structures of H167A–*3F*Glc (PDB code 3PL8 [19]) and H167A–*2F*Glc (PDB code 2IGO [14]) previously. Data processing and scaling were performed with the *XDS* package [36]. Crystal and data collection details for the 14 data sets are given in Table 1 (Glc complexes) and Table 2 (Gal complexes).

### Structure Determination, Model Building and Refinement

Phases were obtained by molecular replacement (PHASER [37]) and Fourier synthesis (FFT, CCP4 [38]). Crystallographic refinement was performed with REFMAC5 [39], and included anisotropic scaling, calculated hydrogen scattering from riding hydrogen atoms, and atomic displacement parameter refinement using the translation, libration, screw-rotation (TLS) model. The TLS models were determined using the TLS Motion Determination server (TLSMD [40]). Corrections of the models were done manually based on σ_A_-weighted 2F_o_-F_c_ and F_o_-F_c_ electron-density maps. The R_free_ reflection sets were kept throughout refinement. All model building was performed with the program O [41] and Coot [42]. Model refinement statistics are given in Table 1 (Glc complexes) and Table 2 (Gal complexes), and the unbiased electron-density maps around the ligand is shown in the Figure S1 (Glc complexes) and Figure S2 (Gal complexes). The electron densities shown were calculated using model phases originating from models that had never contained the ligand, i.e., prior to modeling the ligands, and contoured at the 0.8σ level. The atomic coordinates and structure factors have been deposited with the RCSB with the PDB accession codes: H450G_3FGlc_, 4MOE; H450G_2FGlc_, 4MOF; V546C_3FGlc_, 4MOG; V546C_2FGlc_, 4MOH; H450G/V546C_3FGlc_, 4MOI; H450G/V546C_2FGlc_, 4MOJ; H167A_3Fgal_, 4MOK; H167A_2Fgal_, 4MOL; H450G_3Fgal_, 4MOM; H450G_2Fgal_, 4MOO; V546C_3Fgal_, 4MOP; V546C_2Fgal_, 4MOQ; H450G/V546C_3Fgal_, 4MOR; H450G/V546C_2Fgal_, 4MOS.

## Supporting Information

Figure S1 **Electron density for 3- or 2-fluorinated glucose bound to** ***Tm*** **P2O variants.** Unbiased electron-density maps with final models superimposed for *Tm*P2O variants V546C, H450G and H450G/V546C in complex with 3-fluorinated glucose (panels *a–c*) and 2-fluorinatad glucose (panels *d–f*). The densities for the corresponding complexes for variant H167A have been reported earlier [14], [19]. The electron density was calculated using model phases prior to including the ligand in the model, meaning that the density for the ligand is unbiased by the model. Except for the density for H450G/V546C in complex with *3F*Glc contoured at 1.3*σ*, all electron-density maps were contoured in the range 0.8–1.0*σ*.
(TIF)

Figure S2 **Electron density for 3- or 2-fluorinated galactose bound to** ***Tm*** **P2O variants.** Unbiased electron-density maps with final models superimposed for *Tm*P2O variants V546C, H450G and H450G/V546C in complex with 3-fluorinated galactose (panels *a–c*) and 2-fluorinatad galactose (panels *d–g*). As for Fig. S1, the electron densities are unbiased by the overlaid models. In the case of H167A treated with *3F*Glc, no ligand was bound. The electron-density maps have been contoured at the 0.8σ level. All electron-density maps were contoured in the range 0.8–1.0*σ*.
(TIF)

Figure S3 **Binding of 3- or 2-fluorinated glucose to** ***Tm*** **P2O variants.** (a) H167A–*3F*Glc: This mutant emulates the wild type. It should be noted that the wild type binds the sugar identically as does H167A, but at lower occupancy (unpublished results). H167A binds *3F*Glc in a productive binding mode for C2 oxidation where glucose is stabilized in its *β*-anomeric form.^1^ As expected for the productive 2-oxidation mode, Thr169 Oγ1 is pointing away from the flavin, and the substrate-recognition loop present in the semi-open state. The model has PDB code 3PL8 [19]. The mutated position is 167 and corresponds to the histidine responsible for covalent attachment of FAD. (b) H167A–*2F*Glc: For the competing glucose-binding mode (PDB code 2IGO) [14], glucose is bound as the β-anomer in position for 3-oxidation and the substrate-recognition loop adopts an open conformer with less optimal active-site packing. (c) V546C–*3F*Glc: The *β*-anomeric form of glucose is stabilized for oxidation at C2. As expected for the productive 2-oxidation mode, Thr169 Oγ1 is pointing away from the flavin, and the substrate-recognition loop present in the semi-open state. For clarity, the covalent link between FAD and His167 is not shown, which also applies to panels *d–h*. The mutated position is 546. (d) V546C–*2F*Glc: The competing glucose 3-oxidation mode is identical to that of H167A with the *β*-anomer of glucose oriented for oxidation at C3. The substrate-recognition loop in its fully open conformation and Thr169 pointing away from the flavin. (e) H450G–*3F*Glc: The variant H450G binds *3F*Glc in the productive 2-oxidation mode, identical to that of H167A (see panel *a*) [19], as well as the wild type (unpublished results), and V546C (see panel *c*). Compared with H167A and V546C, which retain His450, the backbone at position 450 in H450G is relaxed by the His450→Gly replacement. The mutated position is 450. (f) H450G–*2F*Glc: The competing glucose-binding mode represented by *2F*Glc shows the sugar stabilized by active site as the α-anomer. The axial O1 of the *2F*Glc *α*-anomer is stabilized specifically by Asp452 Oδ2 and Thr169 Oγ1. (g) H450G/V546C–*3F*Glc: The variant H450G/V546C binds *3F*Glc in the productive glucose 2-oxidation mode; identical to the *3F*Glc complexes of the other variants (panels *a,c,e*). As for H450G, the backbone at position 450 in is relaxed by the His450→Gly replacement. In this mutant both position 450 and 546 have been mutated. (h) H450G/V546C–*2F*Glc: as for H450G, *2F*Glc is stabilized by the H450G/V546C active site in its *α*-anomeric state with identical protein-sugar interactions.
(TIF)

Figure S4 **Binding of 3- or 2-fluorinated galactose to** ***Tm*** **P2O variants.** (a) H167A–*3F*Gal: No ligand is bound, and the substrate-recognition loop assumes the fully closed conformation that is incompatible with sugar binding during the reductive half-reaction. This conformer has previously been observed in *Tm*P2O without bound sugar (PDB code 1TT0 [12]). (b) H167A–*2F*Gal: The competing binding mode for 2-oxidation of galactose by H167A (and probably the wild type) corresponds to the C1-oxidation mode, *i.e.,* the sugar is neither bound in the 2-oxidation or 3-oxidation mode, but positions C1 of β-anomeric galactose in position for oxidation. The sugar is stabilized in the *β*-anomeric configuration showing the substrate-recognition loop in the semi-open conformation and the Thr169 side chain hydroxyl group pointing away from the flavin N(5)/O(4) locus. (c) V546C–*3F*Gal: Productive binding mode for 2-oxidation of galactose where *3F*Gal is bound in the expected orientation and stabilized in the *β*-anomeric form. The substrate-recognition loop is in the semi-open state and Thr169 Oγ1 points away from the flavin. The axial C4 hydroxyl assumes the same position as the axial O1 in the *α*-anomer of *2F*Glc (competing glucose-binding mode for 3-oxidation), receiving stabilizing interactions from Asp452 Oδ2 and Thr169 Oγ1. The overall binding mode is identical to that observed in the 2-oxidation mode for *3F*Glc (see Fig. S1 *a–d*). (d) V546C–*2F*Gal: As for H167A (wild-type mimic; panel *b*), the 1-oxidation binding mode of galactose is preferred by V546C. (e) H450G–*3F*Gal: *3F*Gal is present as the *β*-anomer bound in the expected galactose 2-oxidation mode. Except for two possible additional hydrogen bonds, the binding is identical to that in V546C (panel *c*). (f) H450G–*2F*Gal: As observed for the H450G-*2F*Glc complex (Fig. S1 *g*), *2F*Gal is stabilized in its α-anomer with the axial C1 hydroxyl group stabilized by Asp452 Oδ2 and Thr169 Oγ1. The galactose molecule is positioned for 3-oxidation, representing the competing binding mode for 2-oxidation. The pyranose ring appears somewhat strained. (g) H450G/V546C–*3F*Gal: The binding mode of *3F*Gal is identical to that observed for V546C (panel *c*). (h) H450G/V546C–*2F*Gal: As for H450G (panel *f*), the sugar is bound as the *α*-anomer.
(TIF)
