# Supplementary figures and images for: Structural Basis for Binding of Fluorinated Glucose and Galactose to Trametes multicolor Pyranose 2-Oxidase Variants with Improved Galactose Conversion
Source: PLoS One. 2014 Jan 21;9(1):e86736. doi: 10.1371/journal.pone.0086736 (PMC3897772; doi:10.1371/journal.pone.0086736)

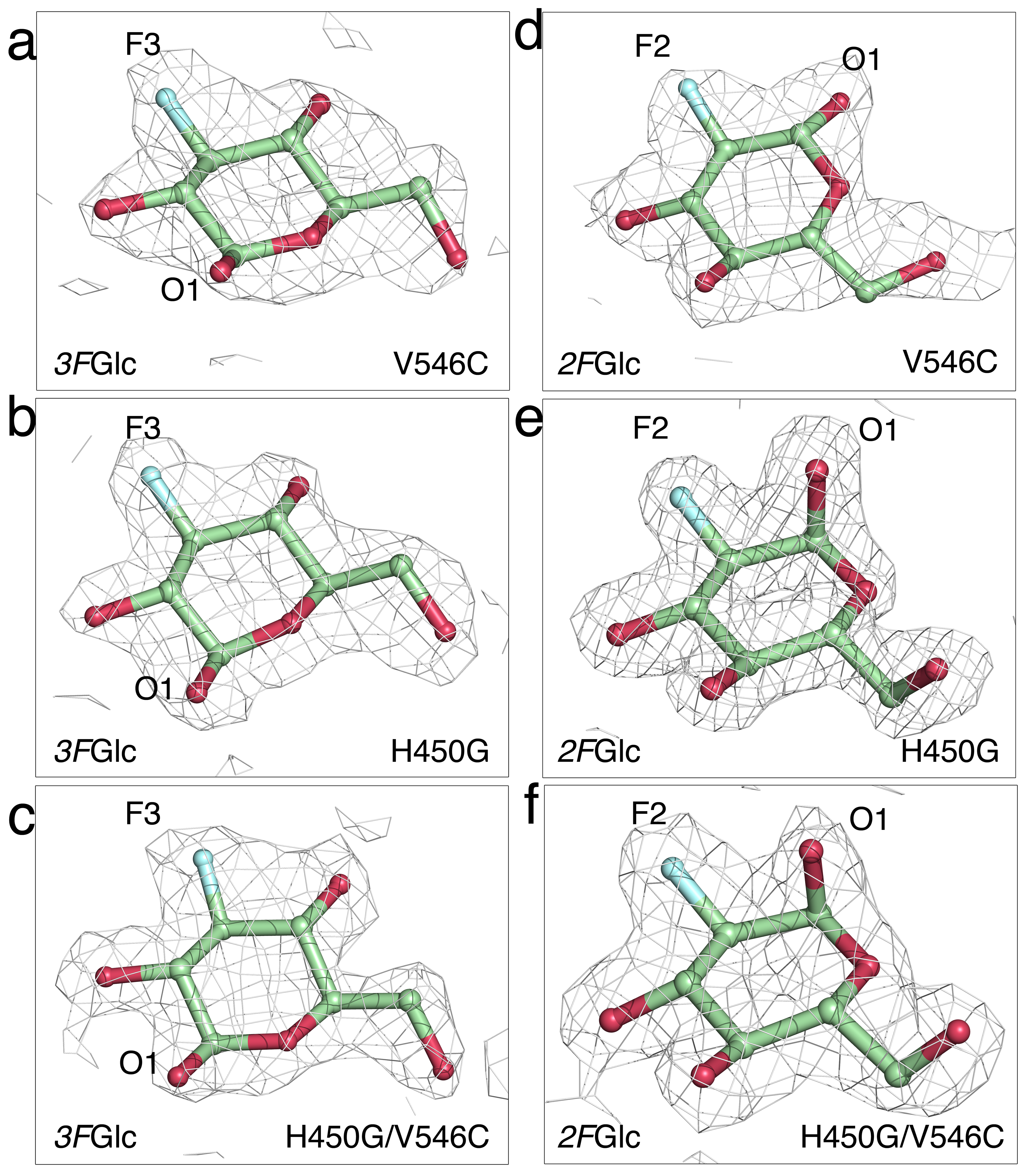

Supplement: Figure S1 — Electron density for 3- or 2-fluorinated glucose bound to Tm P2O variants. Unbiased electron-density maps with final models superimposed for TmP2O variants V546C, H450G and H450G/V546C in complex with 3-fluorinated glucose (panels a–c) and 2-fluorinatad glucose (panels d–f). The densities for the corresponding complexes for variant H167A have been reported earlier [14], [19]. The electron density was calculated using model phases prior to including the ligand in the model, meaning that the density for the ligand is unbiased by the model. Except for the density for H450G/V546C in complex with 3FGlc contoured at 1.3σ, all electron-density maps were contoured in the range 0.8–1.0σ. (TIF) [file pone.0086736.s001.tif]

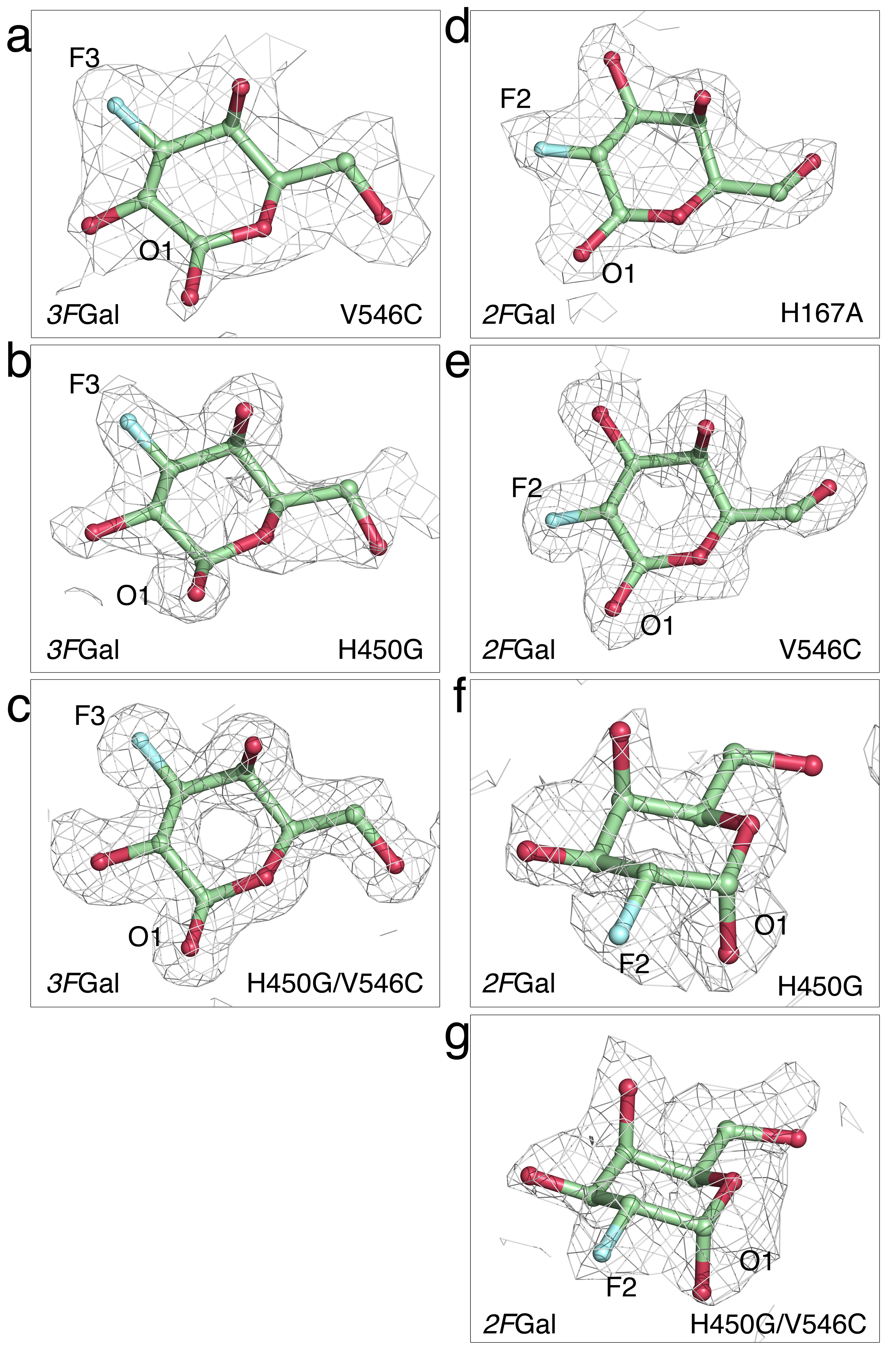

Supplement: Figure S2 — Electron density for 3- or 2-fluorinated galactose bound to Tm P2O variants. Unbiased electron-density maps with final models superimposed for TmP2O variants V546C, H450G and H450G/V546C in complex with 3-fluorinated galactose (panels a–c) and 2-fluorinatad galactose (panels d–g). As for Fig. S1, the electron densities are unbiased by the overlaid models. In the case of H167A treated with 3FGlc, no ligand was bound. The electron-density maps have been contoured at the 0.8σ level. All electron-density maps were contoured in the range 0.8–1.0σ. (TIF) [file pone.0086736.s002.tif]

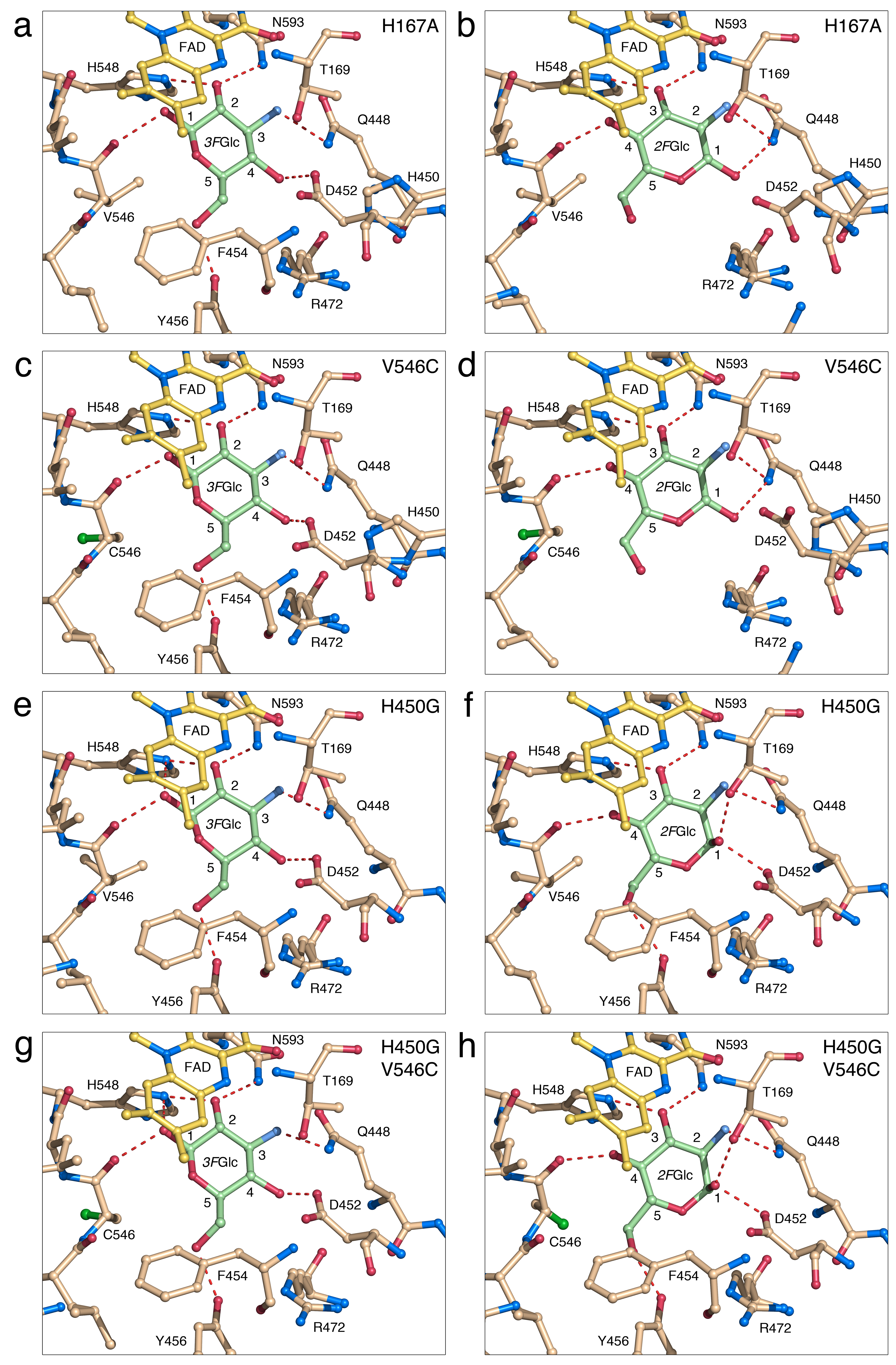

Supplement: Figure S3 — Binding of 3- or 2-fluorinated glucose to Tm P2O variants. (a) H167A–3FGlc: This mutant emulates the wild type. It should be noted that the wild type binds the sugar identically as does H167A, but at lower occupancy (unpublished results). H167A binds 3FGlc in a productive binding mode for C2 oxidation where glucose is stabilized in its β-anomeric form.1 As expected for the productive 2-oxidation mode, Thr169 Oγ1 is pointing away from the flavin, and the substrate-recognition loop present in the semi-open state. The model has PDB code 3PL8 [19]. The mutated position is 167 and corresponds to the histidine responsible for covalent attachment of FAD. (b) H167A–2FGlc: For the competing glucose-binding mode (PDB code 2IGO) [14], glucose is bound as the β-anomer in position for 3-oxidation and the substrate-recognition loop adopts an open conformer with less optimal active-site packing. (c) V546C–3FGlc: The β-anomeric form of glucose is stabilized for oxidation at C2. As expected for the productive 2-oxidation mode, Thr169 Oγ1 is pointing away from the flavin, and the substrate-recognition loop present in the semi-open state. For clarity, the covalent link between FAD and His167 is not shown, which also applies to panels d–h. The mutated position is 546. (d) V546C–2FGlc: The competing glucose 3-oxidation mode is identical to that of H167A with the β-anomer of glucose oriented for oxidation at C3. The substrate-recognition loop in its fully open conformation and Thr169 pointing away from the flavin. (e) H450G–3FGlc: The variant H450G binds 3FGlc in the productive 2-oxidation mode, identical to that of H167A (see panel a) [19], as well as the wild type (unpublished results), and V546C (see panel c). Compared with H167A and V546C, which retain His450, the backbone at position 450 in H450G is relaxed by the His450→Gly replacement. The mutated position is 450. (f) H450G–2FGlc: The competing glucose-binding mode represented by 2FGlc shows the sugar stabilized by active site as [file pone.0086736.s003.tif]

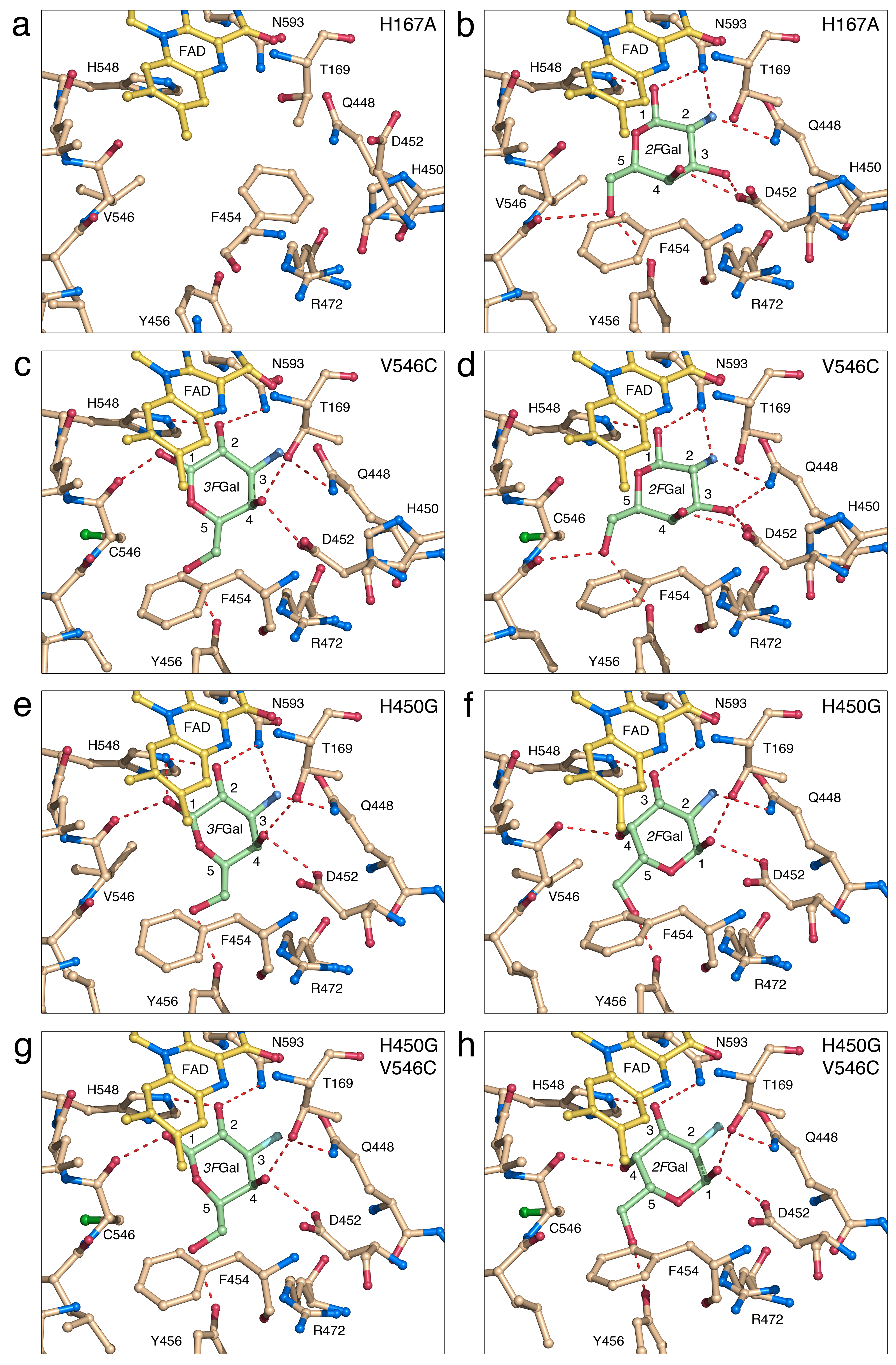

Supplement: Figure S4 — Binding of 3- or 2-fluorinated galactose to Tm P2O variants. (a) H167A–3FGal: No ligand is bound, and the substrate-recognition loop assumes the fully closed conformation that is incompatible with sugar binding during the reductive half-reaction. This conformer has previously been observed in TmP2O without bound sugar (PDB code 1TT0 [12]). (b) H167A–2FGal: The competing binding mode for 2-oxidation of galactose by H167A (and probably the wild type) corresponds to the C1-oxidation mode, i.e., the sugar is neither bound in the 2-oxidation or 3-oxidation mode, but positions C1 of β-anomeric galactose in position for oxidation. The sugar is stabilized in the β-anomeric configuration showing the substrate-recognition loop in the semi-open conformation and the Thr169 side chain hydroxyl group pointing away from the flavin N(5)/O(4) locus. (c) V546C–3FGal: Productive binding mode for 2-oxidation of galactose where 3FGal is bound in the expected orientation and stabilized in the β-anomeric form. The substrate-recognition loop is in the semi-open state and Thr169 Oγ1 points away from the flavin. The axial C4 hydroxyl assumes the same position as the axial O1 in the α-anomer of 2FGlc (competing glucose-binding mode for 3-oxidation), receiving stabilizing interactions from Asp452 Oδ2 and Thr169 Oγ1. The overall binding mode is identical to that observed in the 2-oxidation mode for 3FGlc (see Fig. S1 a–d). (d) V546C–2FGal: As for H167A (wild-type mimic; panel b), the 1-oxidation binding mode of galactose is preferred by V546C. (e) H450G–3FGal: 3FGal is present as the β-anomer bound in the expected galactose 2-oxidation mode. Except for two possible additional hydrogen bonds, the binding is identical to that in V546C (panel c). (f) H450G–2FGal: As observed for the H450G-2FGlc complex (Fig. S1 g), 2FGal is stabilized in its α-anomer with the axial C1 hydroxyl group stabilized by Asp452 Oδ2 and Thr169 Oγ1. The galactose molecule is positioned for 3-oxidation, representing the comp [file pone.0086736.s004.tif]
